# lncRNA Gene Signatures for Prediction of Breast Cancer Intrinsic Subtypes and Prognosis

**DOI:** 10.3390/genes9020065

**Published:** 2018-01-26

**Authors:** Silu Zhang, Junqing Wang, Torumoy Ghoshal, Dawn Wilkins, Yin-Yuan Mo, Yixin Chen, Yunyun Zhou

**Affiliations:** 1 Department of Computer and Information Science, University of Mississippi, Oxford, MS 38677, USA; szhang6@go.olemiss.edu (S.Z.); tghoshal@go.olemiss.edu (T.G.); dwilkins@olemiss.edu (D.W.); ychen@cs.olemiss.edu (Y.C.); 2 Department of Surgery, Ruijin Hospital, Shanghai Jiao Tong University School of Medicine, Shanghai 20025, China; wangjunqingmd@163.com; 3 Department of Pharmacology and Toxicology, University of Mississippi Medical Center, Jackson, MS 39216, USA; ymo@umc.edu; 4 Department of Data Science, John D. Bower School of Population Health, University of Mississippi Medical Center, Jackson, MS 39216, USA

**Keywords:** lncRNA, intrinsic subtypes, feature selection, breast cancer

## Abstract

**Background:** Breast cancer is intrinsically heterogeneous and is commonly classified into four main subtypes associated with distinct biological features and clinical outcomes. However, currently available data resources and methods are limited in identifying molecular subtyping on protein-coding genes, and little is known about the roles of long non-coding RNAs (lncRNAs), which occupies 98% of the whole genome. lncRNAs may also play important roles in subgrouping cancer patients and are associated with clinical phenotypes. **Methods:** The purpose of this project was to identify lncRNA gene signatures that are associated with breast cancer subtypes and clinical outcomes. We identified lncRNA gene signatures from The Cancer Genome Atlas (TCGA )RNAseq data that are associated with breast cancer subtypes by an optimized 1-Norm SVM feature selection algorithm. We evaluated the prognostic performance of these gene signatures with a semi-supervised principal component (superPC) method. **Results:** Although lncRNAs can independently predict breast cancer subtypes with satisfactory accuracy, a combined gene signature including both coding and non-coding genes will give the best clinically relevant prediction performance. We highlighted eight potential biomarkers (three from coding genes and five from non-coding genes) that are significantly associated with survival outcomes. **Conclusion:** Our proposed methods are a novel means of identifying subtype-specific coding and non-coding potential biomarkers that are both clinically relevant and biologically significant.

## 1. Introduction

Molecularly targeted therapies significantly contribute to efforts toward personalized approaches for the treatment of breast cancer, one of the most aggressive and prevalent diseases in women [1]. In 2017, an estimated 252,710 new cases of invasive breast cancer are expected to be diagnosed in women in the U.S., along with 63,410 new cases of non-invasive (in situ) breast cancer [2]. However, breast cancer is one of the most heterogeneous cancer with many subtypes, and the treatment strategy is very different for different subtypes, even though their prognostic outcomes may be similar. 

Clinically, breast cancer has main four subtypes: Luminal A, Luminal B, Her2 positive, and Basal [3,4,5]. The original methods for grouping subtypes are based on immunohistochemistry (IHC) markers such as ER (estrogen receptor), PR (progesterone receptor), and HER2 (epidermal growth factor receptor-2) [6] status, as well as gene expression profiles and their related pathways from high-throughput microarrays [7]. Identification of molecules from each clinically relevant subtypes is of particular importance for therapeutic decision-making and disease management. However, current classification methods of breast cancer subtypes are now limited to protein-coding genes (PCGs), despite the fact that the non-coding region occupies 98% of the whole genome and plays a regulatory role for PCGs [8,9,10]. Also, no studies reported the prediction of breast cancer subtypes by long non-coding RNAs (>200 nt in length) profiles, although Koboldt et al., [4] used the small, micro-non-coding RNAs (micro-RNA) expressions. 

Recent studies have shown long non-coding RNAs to be involved in breast cancer progression via certain biological mechanisms and to display characteristics typical of cancer subtypes [11,12,13]. Generally, lncRNAs that represent similar subtypes or share similar functionality with their correlated PCGs are associated with patient treatment outcomes and survival in tumorigenesis [14,15,16,17]. However, traditional methods of classifying breast cancer subtypes are based on transcriptomic alterations in PCG regions, ignoring any aberrance within non-coding regions owing to the lack of whole genome maps [18,19]. For example, the PAM50 classifier, derived from microarrays, is the most popular and widely used protein-coding gene signature for subgrouping breast cancer patients in clinical practice [20].

One approach to identify patient subgroups/clusters uses an unsupervised learning method based on expression data. However, the drawback of this method is that it is usually not necessarily related to clinical outcomes. Another approach is the supervised learning method, which aims to identify molecular targets associated with cancer subtypes supervised in terms of clinical outcomes. The most famous supervised learning methods are support vector machines (SVMs) [21,22] and random forest algorithms (RFs) [23]. An SVM selects genes with higher weights, and an RF considers a gene important if classification accuracy decreases dramatically when its values in a node of the tree are randomly permuted. However, these machine learning methods that select features associated with different phenotypes are limited in PCGs; little attention has been paid to long non-coding genes. In addition, since a relative lower gene expression than that of PCG often characterizes lncRNA, rendering the detection of comparable and co-existing lncRNA signals a considerable challenge [24,25,26].

To assist comprehensive studies of all types of transcripts and their role in breast tumorigenesis, we propose a novel procedure that will be able to identify cancer subtype-specific biomarkers by a supervised multi-class feature selection method using the 1-Norm Support Vector Machine (SVM) [22] with recursive selection (adapting ideas from SVM-RFE). Our previous study has shown that this optimized approach can be used to select significant coding genes across multiple platforms with competitive classification accuracy and higher performance in prognosis evaluation, compared with the clinically accepted gene signature “PAM50” for breast cancer subtype diagnosis [27]. To improve the prediction accuracy, we integrated RNAseq data with PAM50 classifier from microarrays, which is a novel part of our project. We also iteratively selected a smaller number of coding and non-coding transcripts from RNAseq data but got a decent prediction accuracy without the help of PAM50 classifier. We visualized subtypes via an unsupervised dimensional reduction technique, t-Distributed Stochastic Neighbor Embedding (t-SNE) [28]. We evaluated the association of these gene signatures with survival outcomes via a semi-supervised principal component (superPC) method [29]. We finally identified eight potential biomarkers (three from coding genes and five from non-coding genes) that are significantly associated with clinical outcomes. The evaluation results of prognostic performance and unsupervised visualization suggest that lncRNAs will bring forth a new paradigm for classifying breast cancer patient subtypes.

## 2. Materials and Methods

### 2.1. Data Sources and Description

We downloaded TCGA breast cancer (*n* = 1092) RNAseq and clinical data from the UCSC Xena database (http://xena.ucsc.edu/). Among 1092 samples, 839 of them are labeled the intrinsic subtypes, and 253 are missing subtype information. The raw TCGA RNAseq data (Illumina HiSeq 2000 RNA Sequencing platform) was re-processed with UCSC’s Xena Toil [30] using the GENCODE (version 23) transcript annotation database [31] to quantify protein coding (*n* = 19,797) and non-coding transcript (*n* = 40,701) expression, as shown in Figure S1. 

To improve the focus of our lncRNA study for downstream analysis, given that the non-coding transcript expression of RNAseq data contains many small and uncertain transcripts, we filtered out the small and uncertain transcripts but kept transcripts with a minimum length of 200 bp for lincRNA, antisense, sense_intronic, sense_overlapping, processed_transcripted, and processed_pseudogene categories based on GENCODE v23 annotation. We grouped the pseudogene as one type of long non-coding genes.

Each row was mapped to a unique Ensemble ID, and each column mapped to a patient ID. Normal patients or genes with missing data were removed from the original dataset. Each gene expression was divided by its maximum value for fast training.

We downloaded TCGA breast cancer clinical data for univariate and multivariate survival analysis. A summary of clinicopathologic characteristics for TCGA breast cancer patients is given in Table S4.

### 2.2. Supervised Gene Selection Using Recursive 1-Norm SVM Method

We used the 1-Norm SVM as our gene selection method with recursive selection, similar to our previous study [27]. 1-Norm SVM differs from standard 2-Norm SVM in the objective function for optimization, which is

(1) $$\min_{w,b}\{C\sum_{i=1}^{n}\max{\left\{0,1-y_{i}\left(w^{T}x+b\right)\right\}}^{2}+\left(1-C\right){\left|\left|w\right|\right|}_{1}\}$$

where ${\left|\left|w\right|\right|}_{1}=\sum_{k}\left|w_{k}\right|$ is the 1-Norm of the weight vector, while 2-Norm SVM has the form of $\sum_{k}{w_{k}}^{2}$. After the classifier is fitted, the norm of the weight reflects the importance of its corresponding feature in classification. Both 1-Norm and 2-Norm SVM have the ability to select useful features, but the difference between their objective functions results in the former with much fewer non-zero-weight features than the later [22]. The parameter *C* in (1) controls the tradeoff between *loss*, i.e., prediction error of the model, and *penalty*, i.e., the complexity of the model. Fewer features will be selected by setting a smaller *C* with the sacrifice of prediction accuracy. In the context of cancer classification using gene expression data, each tissue sample is an example and with genes as features. After fitting a 1-Norm SVM classifier, non-zero-weight genes will be selected.

**Figure 1:**
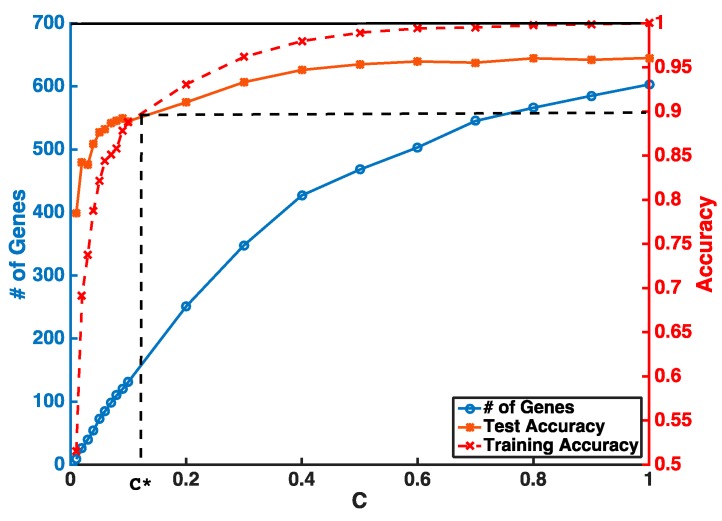
An example of a gene selection curve for the selection from all coding genes.

Instead of setting *C* to be super small in one step, our feature selection has two iterations to obtain a stable smaller gene list. The first step is to guarantee a higher and stable prediction accuracy threshold (i.e., *θ* = 0.9) without considering the number of selected genes. In the second step, we gradually reduce *C* to obtain a smaller number of genes from features selected by the first iteration. An example of a gene selection curve is shown in Figure 1. The training accuracy is about 1 when *C* is close to 1. As *C* becomes smaller, both training and testing accuracies decrease. We stop decreasing *C* at C*, when training accuracy reaches the threshold *θ* and use C* to select the pool of genes for the next iteration. Our experiments show that, even with two iterations of selection, we can select our desired number of genes without sacrificing much of the prediction accuracy.

However, SVM is intrinsically a binary classifier, and when extended to multiclass settings using a strategy of “one vs. the rest”. One classifier will be learned for each class considered as positive with the rest as negative. Genes with non-zero weights in a specific classifier reflect their association with the corresponding positive subtype(s) of that classifier. The advantage of this customized multiclass classification strategy is that we can output the most represented genes and their associated specific breast cancer subtype at the same time. Importantly, the selection procedure is independent of each feature, and the prediction results will not be influenced by each other when different sources of data are integrated together. Our experiments showed that most of the selected genes were associated with only one subtype, and a few genes were associated with a couple.

### 2.3. Breast Cancer Subtype Classification and Prediction Evaluation

The classification accuracies for these selected genes were evaluated by both supervised and unsupervised approaches. In the supervised setting, we used the 2-Norm SVM as our classification algorithm to predict breast cancer subtypes (Basal, Her2, LumA, and LumB) for our selected genes. The prediction accuracies were estimated by 10-fold cross-validation. Any classifier can be used for this purpose. Considering the fact that genes are selected with a linear model and 2-Norm SVM is a state-of-the-art linear classifier, we expect 2-Norm SVM to have better performance than other classifiers.

In the unsupervised setting, we used t-SNE proposed by Maaten and Hinton to reduce the data dimension to 2 for visualization and to compare the clustering effect of selected genes. t-SNE has outperformed other unsupervised dimensionality reduction methods, such as PCA (principle component analysis) and LLE (locally linear embedding), because of its capability of conserving local structure and resolving the “crowding problem” [28]. If these genes are able to predict cancer subtypes, we will expect to see different clusters of these patients through graphic visualization.

### 2.4. Prognosis Evaluation for Selected Gene Signature

Since patients with different cancer subtypes show different treatment responses and survival outcomes, we used a prognostic model to evaluate the performance of the selected gene signature. A semi-supervised principal component algorithm (superPC) [32] was used to evaluate patients’ overall survival and recurrence-free survival outcomes for selected genes. Patients without survival information were removed from further evaluation. Univariate and multivariate Cox proportional models were used to test the association of the risk scores of patients predicted by selected genes with overall survival and recurrence-free survival outcomes.

Kaplan–Meier plots and log-rank test statistics were used to visualize the high- and low-risk groups. The cutoff of the high- and low-risk group was determined by the density distribution of the derived risk score from superPC. We clustered risk groups via the expectation–maximization (EM) algorithm for the normal mixture modeling method by mclust [33].

## 3. Results

### 3.1. Gene Selection and Breast Cancer Subtype Classification

The prediction accuracies for distinguishing subtypes were evaluated by 10-fold cross-validation using a 2-Norm SVM classifier. Since the PAM50 gene signature, which was initially derived from a microarray assay, has been widely used in clinical practice for breast cancer subtype prediction [20], we first tested our method on the PAM50 gene signature from the TCGA microarray dataset. The prediction accuracy reaches 90.9% for PAM50, which suggests that our method is reliable. To investigate whether integrating non-coding genes can improve subtype classification accuracy, we integrated all features including both coding and non-coding genes from RNASeq data with the PAM50 classifier. With the same method, we can improve prediction accuracy to 95% based on cross-validation. This suggests that adding more features from RNAseq data will improve prediction accuracy compared with the traditional microarray platform alone. Therefore, our feature selection method has a competitive strength in selecting most characteristic features with good performance across different platforms and can be used for integrative analysis on other types of “omics” data, such as methylation and copy number variation experiments.

Table 1 shows the number of selected features and subtype prediction accuracies for coding, long non-coding genes, and their mixed genes from RNAseq data with the help of integrating PAM50 signals. We can see that the prediction accuracy of lncRNAs (95.3%) can be as good as PGCs (95.5%) with a similar number of selected features (lncRNAs, *n* = 85; PGCs, *n* = 100). When pooling coding and non-coding genes from RNAseq data together, we selected 106 genes (43 from PCGs, and 63 from lncRNAs) with a slightly higher prediction accuracy (95.8%). This suggested prediction accuracy performs relatively better when combined coding and non-coding genes. The detailed gene annotations and their mapped subtypes (after excluding “normal-like” subtypes) from PCGs, lncRNAs, and the mixed gene signatures are shown in Table S1.

However, microarray and RNAseq data for a patient may not necessarily be available at the same time, and more RNAseq data will be generated in the future since this approach provides more information compared with the microarray. Thus, we applied our method to RNAseq data to select features from coding and non-coding genes. To ensure that the results of a selected number of genes are comparable to PAM50, we iteratively selected 50 features from coding, non-coding, and all pooled genes.

When selecting a small number of genes (~50) from a large pool (>20 k), we applied a recursive strategy as described in the method to improve stability and performance [4]. In the first iteration, we tuned the parameter C to obtain a larger number of features (i.e., lncRNAs *n* = 466) with a good training accuracy of *θ* = 0.9 as the cutoff. In the second iteration, we shrank the number of features to 50 from the gene pool selected from Iteration 1 and measured the prediction accuracy based on 10-fold cross-validation. We then performed the same strategy on coding, non-coding, and “all” genes. We then further excluded smaller and uncertain features from the 50 non-coding features selected in Iteration 2 but emphasized that long non-coding genes belong to the six pre-defined categories with a length of >200 bp, as described above.

As shown in Table 2, we finally selected 50 PCGs, 29 lncRNAs, and 36 mixed genes (17 coding, and 19 long non-coding genes) in two iterations. The prediction accuracies of the three gene signatures were close to each other, but the “all” gene signature was slightly better than the others (88.5%). This observation suggests that non-coding genes are as essential as coding genes on disease diagnosis and subtype classifications, but the combined list from both will give the best performance, which makes sense practically.

Compared with the results in Table 1, we selected a smaller number of independent features from the RNAseq data, although the prediction accuracy dropped slightly from 95.8 to 88.5%, taking the 36 genes as an example. Clinically, we want a tradeoff between a smaller number of genes and fewer prediction errors, because fewer genes will decrease diagnostic expenses. We think an 88.5% accuracy is acceptable, but we can increase the accuracy by selecting more features. The derived gene signatures and their predicted subtypes can be found in Table S2. Results suggested (1) that most selected genes are associated with only one subtype and a few genes associated with more than one and (2) that more genes were selected in the LumA and LumB subtypes. This is because LumA and LumB are the most confusing classes to distinguish, so more genes are needed.

### 3.2. Visualization of Breast Cancer Subtypes

**Figure 2:**
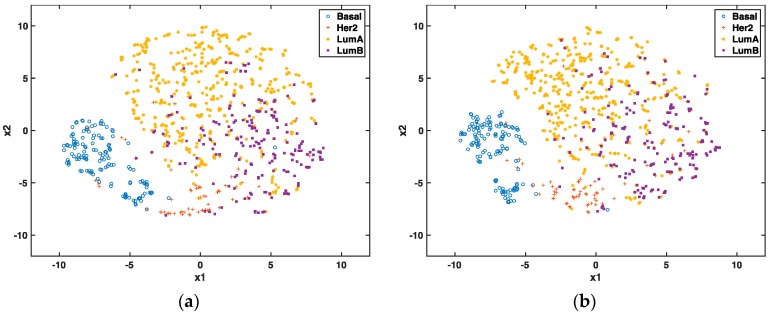

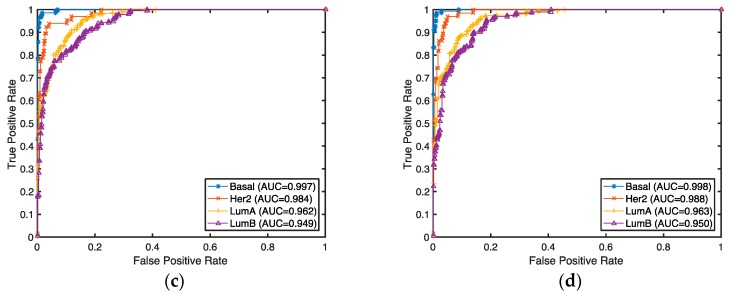
Visualization of breast cancer subtypes using selected 29 non-coding (**a**) and 36 “all” (**b**) gene features for 839 TCGA RNAseq training set; the sensitivity and specificity of prediction accuracy based on ROC curve for 29 non-coding (**c**) and 36 “all” (**d**) gene features.

We further compared selected coding and long non-coding genes using t-SNE, which is an unsupervised dimensionality technique to visualize breast cancer subtypes. In Figure 2, each point represents a sample and its color denotes its cancer subtype. Note that the subtype information is only used for coloring rather than the optimization process of visualization. The clustering effects of non-coding (Figure 2a) and “all” (Figure 2b) genes are comparable. In both figures, Basal and Her2 are well separated from others, LumA and LumB have some ambiguities in their clusters (which is also true in clinical diagnosis). Figure 2c,d show the sensitivity and specificity of the prediction accuracy based on non-coding and “all” genes, respectively. Results show that the Basal subtype is distinguished well compared with other subtypes. This observation further proves that the long non-coding genes capturing different characters of breast cancer subtypes can be as good as all types of genes. 

### 3.3. Gene Signature Evaluations Based on Clinical Data

Our prognostic evaluations of survival analysis for each gene signature are based on TCGA overall survival and recurrence-free survival outcomes. The prognostic model for each signature was trained via 10-fold cross-validation by the superPC method as described above. Table 3 shows the *R*^2^ statistic and *p*-value of the derived risk score for three gene signatures based on the Cox proportional hazards analysis. The *R*^2^ statistic measures the percentage of variation in survival time, which prefers a larger *R*^2^ statistic. These results suggest that the subtype-specific gene signatures perform well for prognostic prediction, but the 36 combined gene list gives a stable performance for both overall survival (*R*^2^ = 0.023, *p*-value = 0.00000852) and recurrence-free survival (*R*^2^ = 0.025, *p*-value = 0.0000483). Although the 50 coding gene signature exhibited the best performance in overall survival analysis, it could due to other features involved in the model compared with the 36 combined gene list.

**Figure 3:**
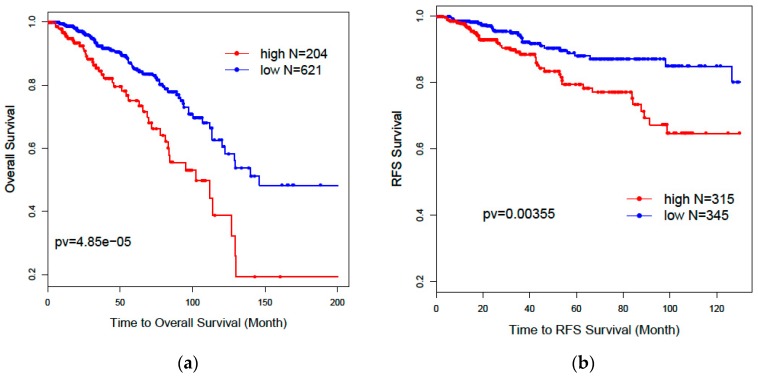
Kaplan–Meier curves and log-rank test *p*-values for overall survival (**a**) and recurrence-free survival (**b**) for the 36 “all” gene signature from 839 RNAseq data.

Figure 3 shows the Kaplan–Meier curves and log-rank test statistics for overall survival (a, OS: *p* = 0.0000485) and recurrence-free survival (b, RFS: *p* = 0.00355) analysis based on high- and low-risk groups derived from the 36 combined gene signature from the 839 RNAseq data. The high- and low-risk groups were separated by the mclust method based on the distribution of risk score predicted by the 36 genes. We can see that the high-risk group has a significantly poor prognosis for both overall survival and recurrence-free survival than the low-risk group. We performed univariate Cox regression analysis for the risk score from the 36 gene signature (HR = 2.71: [1.81, 4.06], *p* = 0.00000110). As shown in Table 4, the multivariate Cox regression model shows that the 36 gene signature can independently predict significantly worse survival outcomes for the high-risk group compared with the low-risk group (HR = 2.3: [1.2, 4.5], p = 0.01), after adjusting for age, race, treatment, tumor stage, and histology.

The superPC method also yielded eight top-ranked genes that are most associated with overall survival from the 36 gene signature, three of which are protein-coding genes (DDX51, SPAG17, and NUMA1) and five of which are lncRNAs (CTD-2616J11.9, RP1-140K8.1, RP11-546K22.1, AC000095.9, and SCGB1D5P). The ranking strategy is based on their univariate Cox proportional hazards scores for each gene and selected only those genes whose Cox coefficient from the proportional hazards model exceeds the absolute value of a threshold hold, which was estimated by the 10-fold cross-validation from the TCGA 839 RNAseq data. The hazard ratio with a 95% confidence interval and a *p*-value from the univariate Cox regression analysis of overall survival for the eight genes are shown in Table 5. 

### 3.4. Application of Supervised Feature Selection Strategy on Independent TCGA RNASeq Set

Since the above models were trained on 839 samples supervised by labeled subtype information, we applied the identified genes on 253 samples without subtype labels to predict subtypes. We assume the predicted subtypes in the testing set should show clinical correlations with immunohistochemistry markers as PR, ER, and Her2 status if available. The individual patient’s predicted labels based on 29 lncRNAs gene signature and 36 combined gene signature for these samples are shown in Table S3. Results showed that 235 of 253 patients (92.9%) were classified as the same subtypes by the two gene signatures.

To test the association of predicted subtypes and IHC markers, we calculated the proportions for the three categories: ER-/PR-/Her2-, ER-/PR-/Her2+, and ER+/PR+/HER2. An example of subtype relationship with the three genes is shown in Figure S2 described by Dai et al. [34]. As shown in Table 6, the predicted Basal subtype is enriched in the ER-/PR-/Her2- category (92%), which is also known as triple negative breast cancer (TNBC). This is consistent with the clinical conclusion that approximately 90% Basal-like subtypes are TNBC. Moreover, the majority of luminal subtypes, consisting of LumA (80.9%) and LumB (14.9%), are enriched in the ER and PR positive groups. This is also consistent with the clinical observations that the ER-positive group tended to be luminal subtypes [34]. The unsupervised dimensionality technique t-SNE for visualizing breast cancer subtypes for the 253 testing set for 29 lncRNAs and 36 combined genes are shown in Figure S3.

## 4. Discussion

Although many studies have predicted intrinsic subtypes for breast cancer patients and their association with prognosis and treatment responses, none of them have used lncRNAs predicting breast cancer subtypes. Since non-coding genes also play important roles in tumorigenesis but were ignored in their functions. To the best of our knowledge, this is the first attempt to use all types of coding and non-coding features from TCGA RNAseq data for the prediction of breast cancer subtypes.

Feature selection is a critical step in identifying target molecules associated with subtypes and could guide clinical decisions for target therapy. The traditional ways of identifying gene signatures for subtypes are based on expression profiles from microarrays, e.g., the PAM50-gene signature from UNC microarray datasets [35] and the 70-gene signature from Fan et al. [36]. However, the prediction accuracy is limited because the gene expression levels vary significantly across different cohort studies, even using the same platform. It is going to be even more challenging if it involves the different platform from different experiments. Therefore, in this project, we developed a supervised feature selection procedure that allows users to select different types of features by integrating different experiment platforms. Our results showed that prediction accuracy could be significantly improved to as high as 95% after integrating all types of RNAseq data and PAM50 gene profiles from the microarray in TCGA. Our previous study has shown that the protein-coding gene signature derived from TCGA RNAseq data could be validated in the microarray data of another independent cohort METABRIC [27]. These conclusions suggest that our methods can accurately predict clinical phenotypes through the integration of multiple data resources.

Our results further showed that lncRNAs could independently predict breast cancer subtypes without any help from the PAM50 classifier. Our two-step recursive 1-Norm SVM feature selection method identified a smaller number of lncRNAs (*n* = 29) from 839 subtype-labeled samples but reached a decent predictive accuracy of 87.8%: [87.6%, 88.0%]. A combined gene list (n = 36) from both lncRNAs and PCGs exhibited a slightly improved prediction accuracy of 88.5%: [88.1%, 88.9%], which is an improvement over PCGs or lncRNAs alone. The prognostic risk scores trained by superPC were used to compare the associations of various gene signatures with overall survival (OS) and recurrence-free survival (RFS). The Cox proportional regression analysis for each gene signature risk score showed that the 36-gene combined list had the best performance for both OS and RFS. We also applied the 36 genes to TCGA 253 unlabeled samples to predict their subtypes. Results showed that 92% of the predicted Basal subgroup was associated with TNBC, and 95.8% of the Luminal subgroup (LumA: 80.9% and LumB: 14.9%) was associated with the ER+/PR+ positive group. Since the above findings are consistent with clinical acceptance, our methods are useful and will provide more prediction options for determining cancer subtypes. In addition to identifying the gene signatures for prediction of breast cancer subtypes, we also identified eight new prognostic markers. Among these prognostic biomarkers, three of them are PCGs, and five of them are lncRNAs. These identified lncRNAs were validated in the laboratory through CRISPR technology. Compared with the currently clinically accepted 50 gene signatures, our eight genes will decrease diagnostic expenses and are easy to validate in biological experiments.

We have provided all types of gene signatures as references for further investigation. Many lncRNAs had previously never reported before. This is partly because we have an insufficient knowledge of lncRNAs and many new lncRNAs have been recently updated. Another reason is that most current lncRNA experimental studies focus on the HGNC-database-approved lncRNAs (*n* = 3851), whose functions are relatively clear. Though the majority of non-coding genes (i.e., 40,701 in our study) annotated by GENCODE v23 database are ambiguous, they might be functionally important and so are worth following up on. For example, one of our lncRNA prognostic biomarkers, CTD-2616J11, has also been reported as a significant prognostic marker for gastric cancer [37]. Meanwhile, the three identified PCGs—DDX51, SPAG17, and NUMA1—have been well studied for their associations with cancers. For example, NUMA1 is associated with a BRCA2 mutation in familial breast cancer [38]. DDX51 controls non-small cell lung cancer proliferation by regulating cell cycle progression via multiple pathways. Moreover, SPAG17 is predominantly expressed in cancer-testis antigens and might serve as a target for cancer immunotherapy [39].

Our methods provide a new perspective on the role of lncRNAs in classifying cancer subtypes and predicting cancer prognosis from all 60,498 genes with both coding and non-coding genes. However, we agree that some of the well-known lncRNAs biomarkers, such as HOTAIR and MALAT1, did not show up using our method. The potential reasons are complicated. Biologically, most of their subtype association findings are based on experimental observations from cell lines or mouse models [40], and, due to the heterogeneity of human tumor tissue samples, these lncRNAs signals are not strong enough to significantly differentiate the subtypes from TCGA RNAseq tumor tissue profiles (data not shown). Additionally, the selected gene signatures from the supervised algorithms largely rely on the labels of the training set. The TCGA 839 labeled training set initially derived from coding gene profiles of the microarray. Therefore, it relatively has the potential to pick the lncRNAs with similar signals for the predictors of the microarray.

Compared with other papers analyzing non-coding genes for breast cancer subtypes, we think that the extent of our contribution relies on the following facts: (1) The other papers only focus on the study of functional known long non-coding genes, thereby ignoring the vast majority of long non-coding genes that have unknown functions. Our results provide more information for further biological validation. (2) Although other papers have identified lncRNAs, none of these papers have evaluated the subtype-specific gene signatures from a prognostic perspective, though the prognosis of individual lncRNA has been evaluated [41,42,43]. (3) Our novel integrative method is flexible in balancing the tradeoff of prediction accuracy and the number of selected genes, which will provide biologists with more options. Overall, our unsupervised learning method and prognostic model comparison further confirmed the prediction strength of our identified genes.

Despite its preliminary character, our future efforts could be directed toward developing feature selection and visualization software for any multi-class phenotypes across different “omics” platforms. Biological determinations of these identified biomarkers can be further investigated through experimental validations.

## 5. Conclusions

Our novel feature method identified protein-coding and non-coding gene signatures for the prediction of breast cancer subtypes. Our results show that prediction accuracies are improved after RNAseq is integrated with the PAM50 classifier from the microarray. Our results also showed that a 36-gene subtype-specific signature combining coding and non-coding genes exhibited the best prognostic performance and classification accuracy. We propose that eight important lncRNAs be investigated as potential biomarkers in the future.

## Figures and Tables

**Table 1:** Subtype prediction accuracy for integrative gene signatures with PAM50.

Gene Type	# Of Genes before Selection	# Of Selected Genes from RNAseq	# Of PAM50 Genes from Microarray	Integrative Classification Accuracy (%) ^1^
PCGs	19797	100	22	95.5: [95.1, 95.9]
lncRNAs	40701	85	21	95.3: [94.8, 95.8]
all	60498	106	19	95.8: [95.4, 96.2]

^1^ Classification accuracy was measured by performing 10-fold cross-validation.

**Table 2:** Evaluation of the prediction accuracies from TCGA RNAseq (*n* = 839) selected genes by two-iteration selection.

Gene Type	# Of Genes Selected in Iteration 1	# Of Genes Selected in Iteration 2	Classification Accuracy (%) ^1^
coding	417	50	87.6: [87.2, 88.0]
non-coding	466	29	87.8: [87.6, 88.0]
all	530	36	88.5: [88.1, 88.9]

^1^ Classification accuracy was measured by performing 10-fold cross-validation 10 times.

**Table 3:** Evaluation of prognostic performance for risk scores from various gene signatures.

Gene Type	# Of Genes	Overall Survival		Recurrence-Free Survival	
		*R*^2^	*p*-Value	*R*^2^	*p*-Value
coding	50	0.031	0.000000485	0.018	0.000588
non-coding	29	0.023	0.0000104	0.017	0.000938
all	36	0.023	0.00000852	0.025	0.0000483

**Table 4:** Multivariate Cox regression survival analysis.

	Hazards Ratio (95% Confidence Interval)	*p*-Value
*Risk Score*	2.3: [1.2, 4.5]	0.01 *
*Age at dignosis*	1.0: [1.0, 1.0]	0.0041 *
*Race*		
White	Reference	
Black	1.5: [0.7, 3.0]	0.31
Asian	0.5: [0.1, 3.9]	0.51
*Treatment*		
Untreated or other	Reference	
Chemotherapy	0.6: [0.3, 1.3]	0.20
Radiation therapy	0.5: [0.2, 1.0]	0.06
Hormone therapy	0.4: [0.1, 1.2]	0.09
Radiation & chemotherapy	0.3: [0.1, 0.6]	0.0027 *
Radiation & hormone	0.3: [0.1, 0.8]	0.02 *
*Tumor stage*		
T1	Reference	
T2	1.4: [0.7, 2.8]	0.33
T3	1.4: [0.6, 3.6]	0.45
T4	1.7: [0.6, 5.6]	0.39
*Histology Type*		
Infiltrating Ductal Carcinoma	Reference	
Infiltrating Lobular Carcinoma	1.2: [0.5, 3.2]	0.70
Mucinous Carcinoma	2.6: [0.3, 22.3]	0.83
Mixed Histology	0.9: [0.3, 2.9]	0.37

* Statistical significance (*p*-value < 0.05).

**Table 5:** Important prognostic biomarkers and univariate overall survival analysis.

Gene_Name	Gene_Type	Chrom (Start–End Position)	HR (95% CI)	*p*-Value
DDX51	PCGs	chr12:132136594-132144335	0.90: [0.84, 0.98]	0.009 *
SPAG17	PCGs	chr1: 117953861-118185223	0.94: [0.89, 0.99]	0.027 *
NUMA1	PCGs	chr11: 72002864-72080693	0.91: [0.87, 0.96]	0.0003.5 *
CTD-2616J11.9	lncRNAs	chr19: 51345169-51353293	0.91: [0.87, 0.96]	0.001 *
RP1-140K8.1	lncRNAs	chr6: 3893126-3894292	1.06: [1.00, 1.13]	0.033 *
RP11-546K22.1	lncRNAs	chr8: 51961458-52022974	0.94: [0.89, 0.99]	0.043 *
AC000095.9	lncRNAs	chr22: 19018043-19018916	0.91: [0.85, 0.98]	0.011 *
SCGB1D5P	lncRNAs	chr4: 165517255-165517501	1.03: [1.00, 1.08]	0.067

* Statistical significance (*p*-value < 0.05).

**Table 6:** Evaluation of selected genes by breast cancer subtype classification.

Predicted Subtypes	ER-/PR-/HER2-	ER-/PR-/HER2+	ER+/PR+/HER2
Basal	23 (92%)	0	2 (2.1%)
Her2	1 (4%)	4 (80%)	2 (2.1%)
Luminal A	1 (4%)	1 (20%)	76 (80.9%)
Luminal B	0	0	14 (14.9%)

## Supplementary Materials

The following are available online at http://www.mdpi.com/2073-4425/9/2/65/s1. Figure S1: Non-coding genes distribution; Figure S2: association of subtypes with PR, ER, and Her2 status; Table S1: Integrative gene signatures for coding and non-coding genes from RNAseq and microarray; Table S2: Coding and non-coding gene signatures based on RNAseq data only; Table S3: Predicted subtype for TCGA 253 testing set.
