# Supplementary material for: lncRNA Gene Signatures for Prediction of Breast Cancer Intrinsic Subtypes and Prognosis
Source: Genes (Basel). 2018 Jan 26;9(2):65. doi: 10.3390/genes9020065 (PMC5852561; doi:10.3390/genes9020065)
Supplement: Supplementary file 1 [file genes-09-00065-s001.zip › Supp figures 1108.pdf]

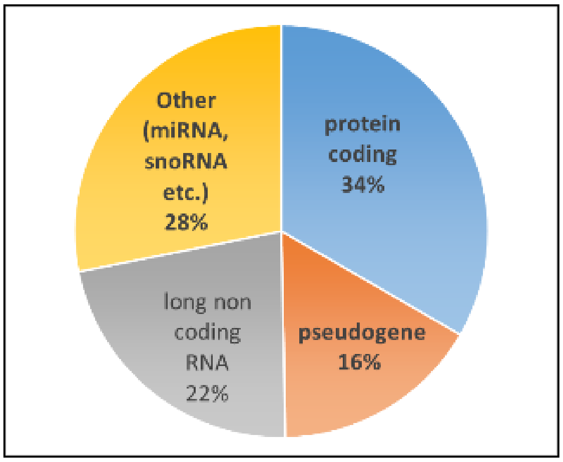

Supp. Fig 1. Transcripts Distribution in GENCODE (v23)

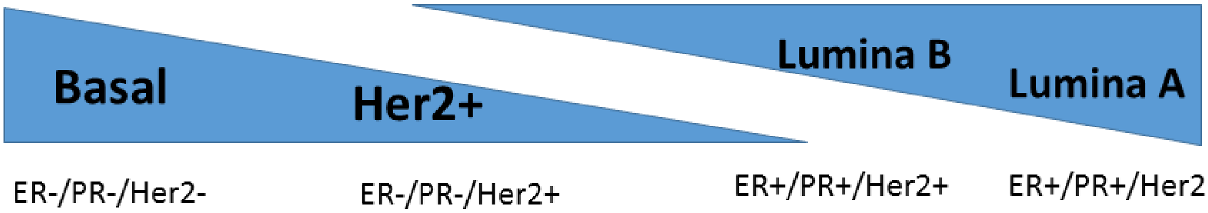

Supp. Fig 2. Relationship of breast cancer intrinsic subtypes and IHC biomarker status

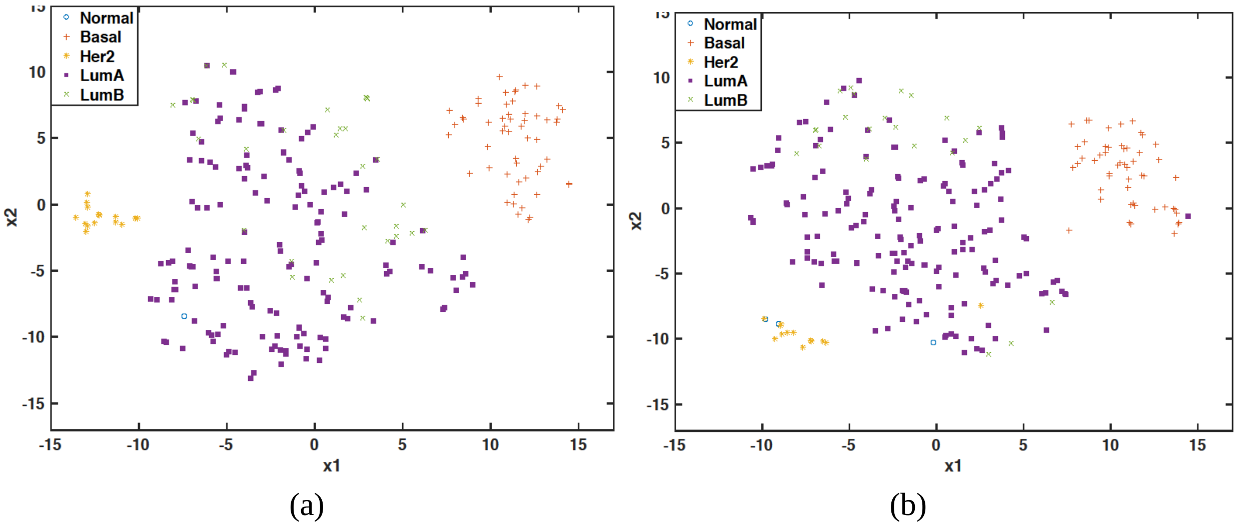

Supp. Fig 3. Visualization of breast cancer subtypes using selected 29 non-coding (a) and 36 all gene features for 253 TCGA RNAseq testing set.
