# Supplementary figures and images for: lncRNA Gene Signatures for Prediction of Breast Cancer Intrinsic Subtypes and Prognosis
Source: Genes (Basel). 2018 Jan 26;9(2):65. doi: 10.3390/genes9020065 (PMC5852561; doi:10.3390/genes9020065)

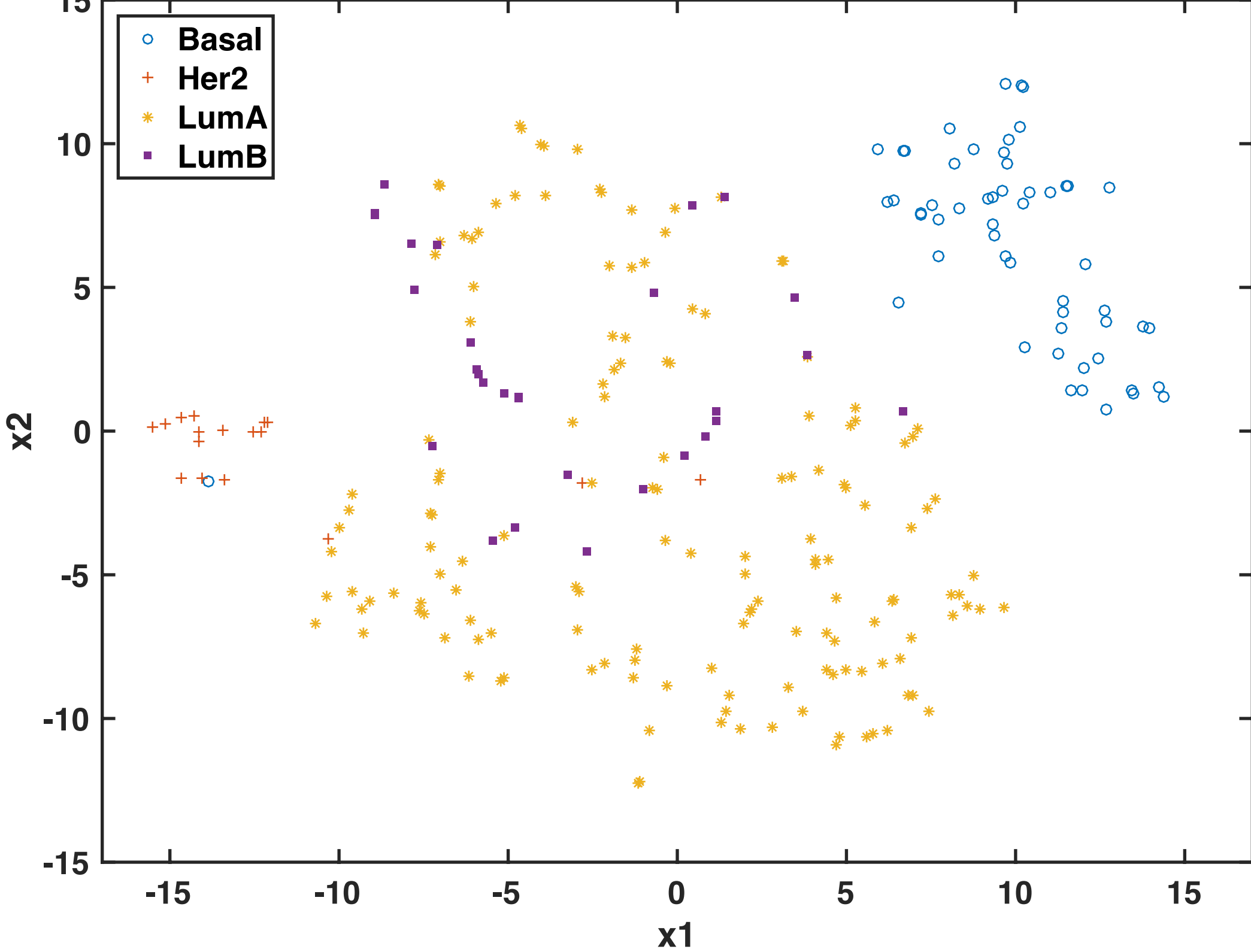

Supplement: Supplementary file 1 [file genes-09-00065-s001.zip › supp.fig3a-nonCoding.pdf]

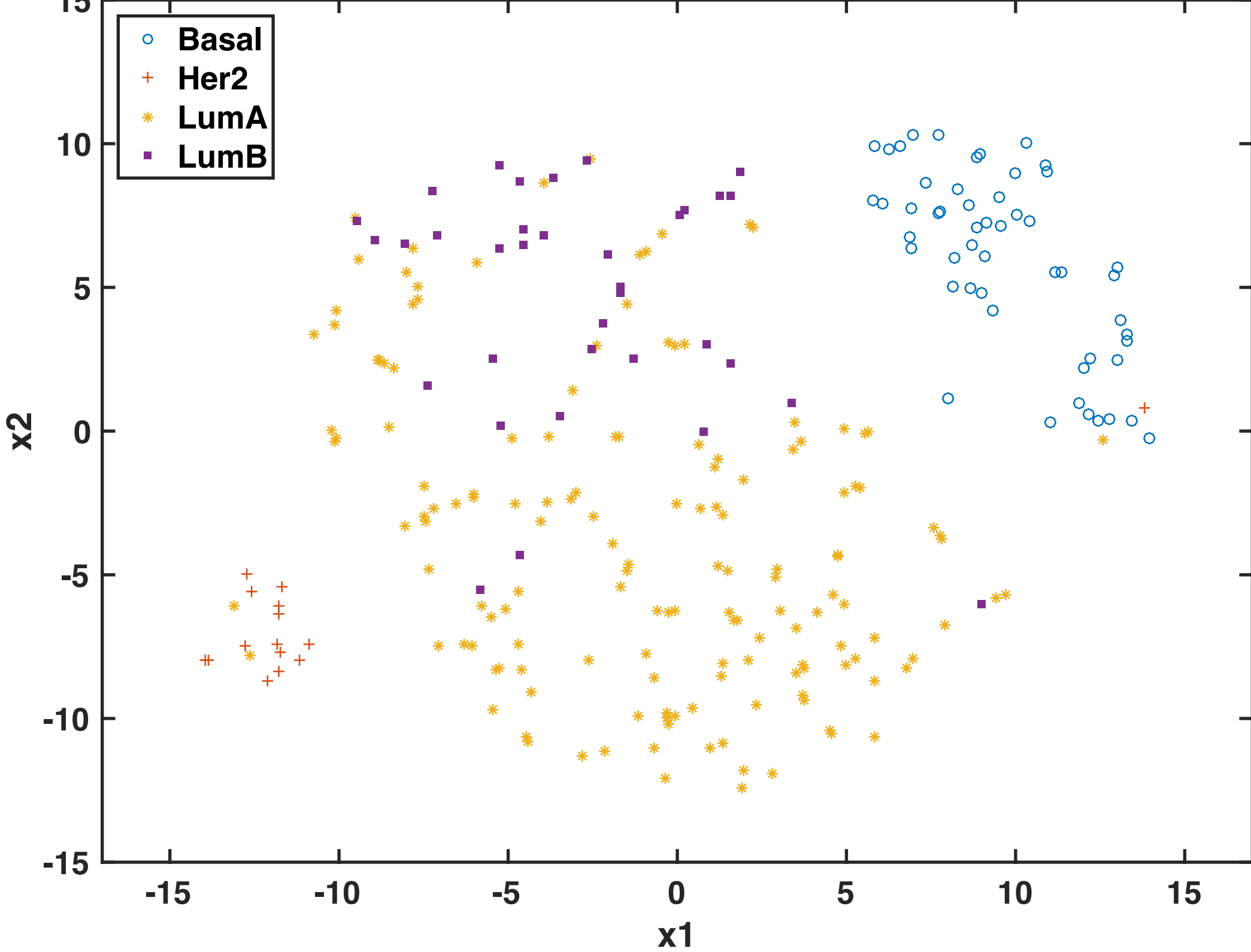

Supplement: Supplementary file 1 [file genes-09-00065-s001.zip › supp.fig3b-all.pdf]
